# Supplementary material for: DMRforPairs: identifying Differentially Methylated Regions between unique samples using array based methylation profiles
Source: BMC Bioinformatics. 2014 May 15;15:141. doi: 10.1186/1471-2105-15-141 (PMC4046028; doi:10.1186/1471-2105-15-141)
Supplement: Additional file 2 — DMRforPairs output for the comparison of A431-MCF7 and NA17018-NA17105. Please start from the HTML files in each folder. Available via the BMC Bioinformatics website. [file 1471-2105-15-141-S2.zip › 1394847754114233_MOESM2_ESM/A431_MCF7/figures/31666.pdf]

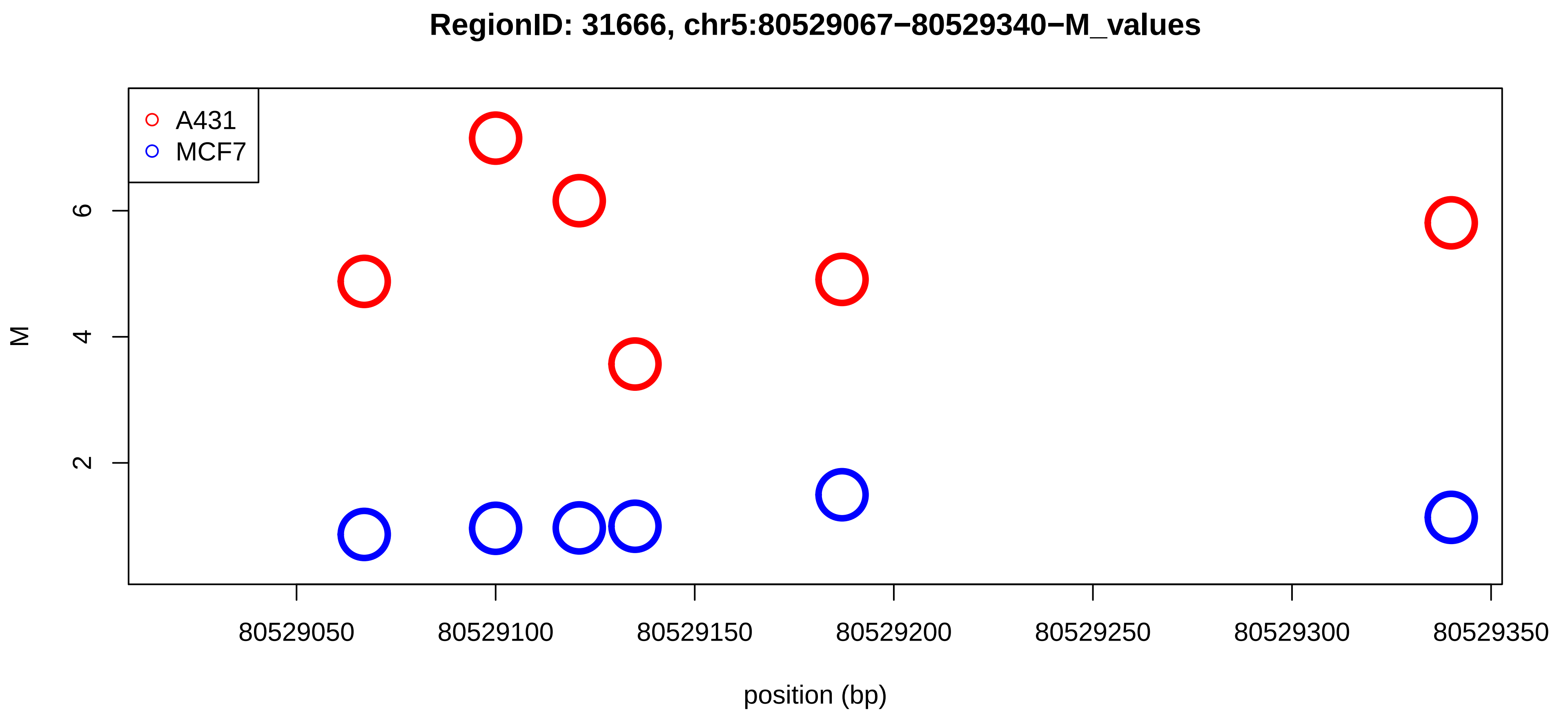
RegionID: 31666, chr5:80529067−80529340−M_values
A431
MCF7
M
2
4
6
80529050
80529100
80529150
80529200
80529250
80529300
80529350
position (bp)

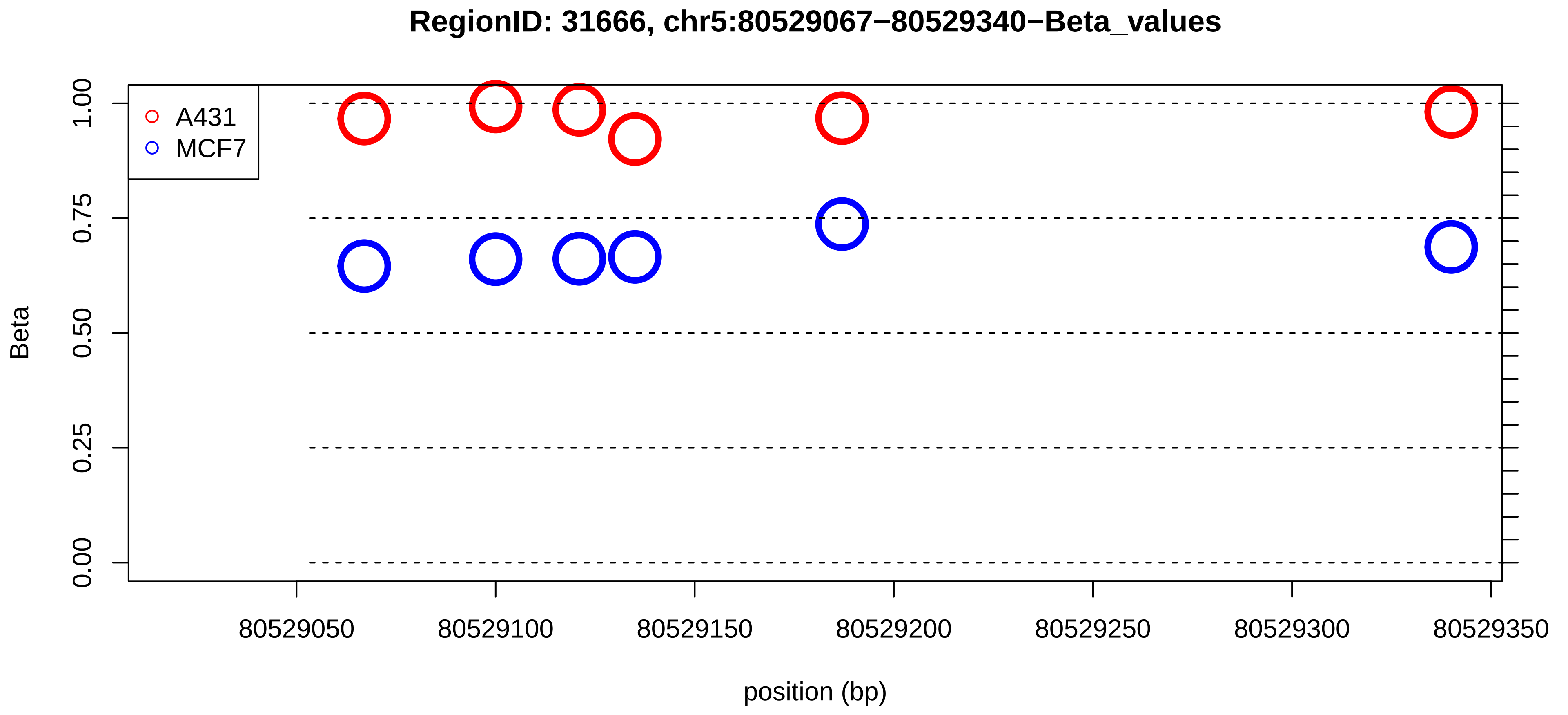
RegionID: 31666, chr5:80529067−80529340−Beta_values
A431
MCF7
Beta
0.00
0.25
0.50
0.75
1.00
80529050
80529100
80529150
80529200
80529250
80529300
80529350
position (bp)

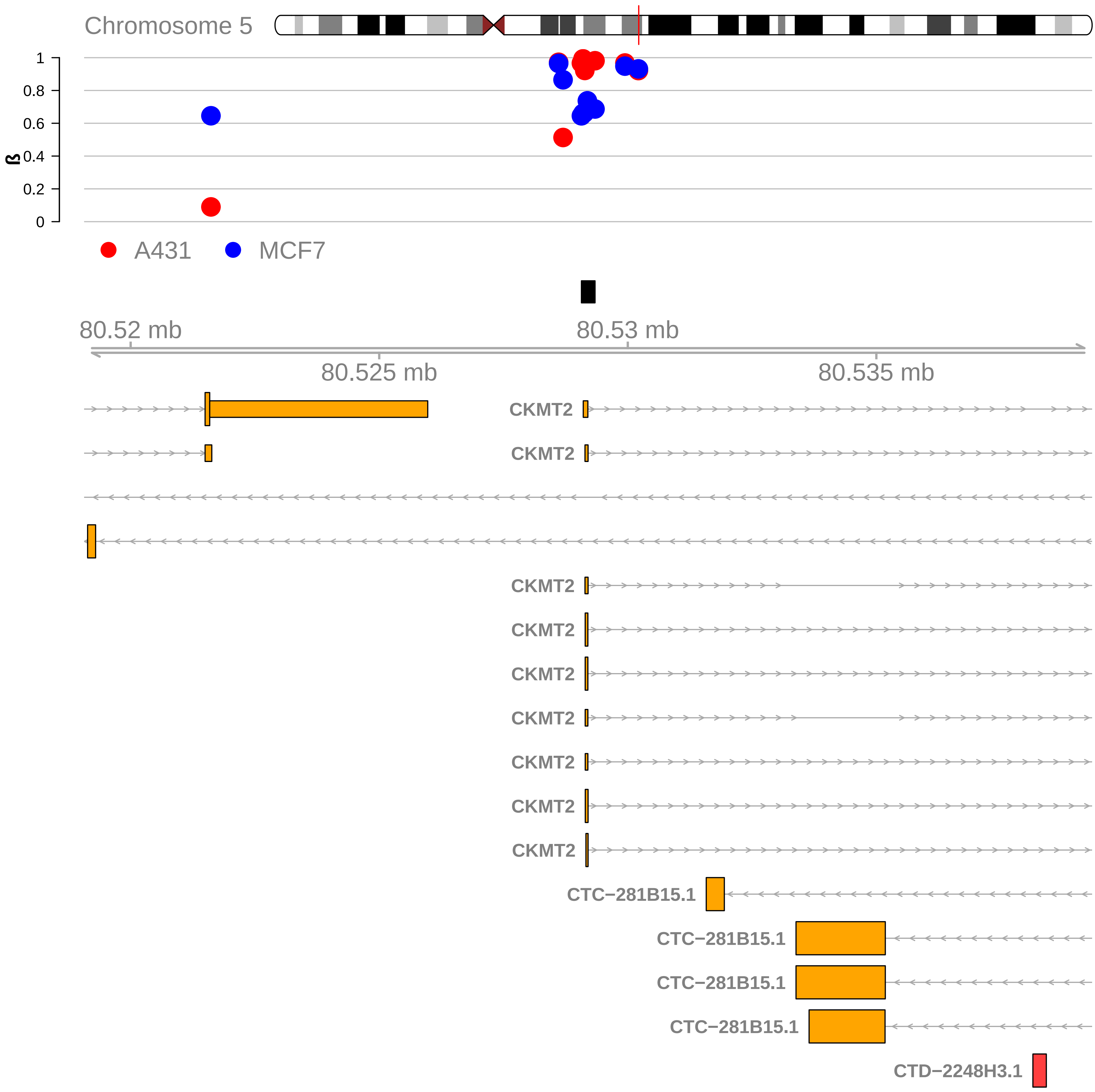

Chromosome 5
ß
1
0.8
0.6
0.4
0.2
0
A431
MCF7
80.52 mb
80.525 mb
80.53 mb
80.535 mb
CKMT2
CKMT2
CKMT2
CKMT2
CKMT2
CKMT2
CKMT2
CKMT2
CKMT2
CTC−281B15.1
CTC−281B15.1
CTC−281B15.1
CTC−281B15.1
CTD−2248H3.1
